# The Medical Council and the Apothecaries' Hall of Ireland

**Published:** 1897-04-17

**Authors:** 


Vol. XXII.—No. 551.]

[April 17, 1897.

### The Medical Council and the Apothecaries' Hall of Ireland.

For the second time in its history of nearly forty years the General Medical Council has come into collision with one of its constituent corporations, and for the second time has been defeated before the highest tribunal to which the question at issue could be referred. The first occasion arose out of the refusal of the Council to recognise the right of the Royal College of Physicians, under an ancient charter, to conduct a qualifying examination in surgery as well as in medicine; and this right, which it only desired to exercise in certain exceptional cases, the College succeeded in establishing. The second occasion has been furnished by the refusal of the Council to appoint assistant examiners in surgery for the purpose of enabling the Apothecaries' Hall of Ireland to confer a complete title to registration; against which refusal the Hall has successfully appealed to the Privy Council. It will be remembered that prior to the Medical Act of 1886 either a medical qualification alone, or a surgical qualification alone, could be registered, but that after 1886 every candidate for entrance into the medical profession was compelled to pass a qualifying examination in medicine, surgery, and midwifery. Certain examining bodies, however, had no power to institute a complete examination as thus defined, and the Act of 1886 provided that bodies so circumstanced might enter into combinations with other bodies in order to form conjoint boards capable of giving complete qualifications; and, further, that if any medical corporation represented to the General Medical Council that it was unable to enter into such a combination, and the Council was satisfied as to its inability to do so on reasonable terms, "it shall be lawful for the General Council from time to time, if they think fit, on the application of such corporation, to appoint any number of examiners to assist at the examinations which are held by such corporation for the purpose of granting any diploma or diplomas conferring on the holders thereof, if they have passed a qualifying examination, the right of registration under the Medical Acts."

The College of Surgeons in Ireland therefore entered into two combinations, and formed one conjoint board with the Dublin College of Physicians, and another with the Apothecaries' Hall of Ireland. The medical portion of the examination conducted by this second conjoint board was unfavourably described by the Inspector and Visitor appointed by the Medical Council; and the Examinations Committee of the Council, in its report to the general body upon the inspections, suggested that the College of Surgeons of Ireland would consult its dignity by withdrawing from the combination. This course the College thought fit to adopt, and gave the necessary notice to the Hall; but, in the meanwhile, the examination did not improve, and was at last reported to the Privy Council by the Medical Council as being "insufficient" to justify registration. On this, the Privy Council intimated that, as the board was about to die a natural death by the withdrawal of the College of Surgeons, there was no necessity for any action. The Hall, however, applied to the Medical Council for the appointment of assistant examiners in surgery; and the Council, mainly on the ground that its dissatisfaction had been with the medical side of the examination and not with the surgical side, exercised the discretion assumed to be implied in the words "if they think fit," and refused the application. The Hall then appealed to the Privy Council, the supreme authority on such questions under the Act, and the Privy Council, after a partial hearing, suggested that some way out of the difficulty might perhaps be found, and deferred its decision. The application of the Hall to the Medical Council was however renewed, and was again refused in the winter session of 1896. On March 22nd the case was finally heard by the Privy Council, the Lords present being the Duke of Devonshire, the President, with Lords Macnaghten, James of Hereford, and Rathmore. Judgment was given on the 9th inst., their Lordships being of opinion that occasion had arisen for the General Council to appoint assistant examiners in surgery for the purpose of qualifying examinations to be held by the Apothecaries' Hall of Ireland, and accordingly directing the Medical Council to appoint them. If this direction were not obeyed the Privy Council would have power under the Act to appoint the assistant examiners itself.

This judgment must be taken to represent the purely legal aspects of the case, with possibly a side glance at the inconveniences which might attend the establishment of a new Irish grievance. The Medical Council, however, will have the satisfaction of having tried to do its duty for the protection of the public, although it has been over-ruled by *force majeure*; and the Dublin Hall, which has had a very narrow escape from extinction as an examining body, will probably take the lesson to heart, and will see that its examiners in medicine do not offend in the way of undue leniency or carelessness in future. Should they do so, the Council will retain the power of reporting "insufficiency," and it would be hardly possible, even for the Privy Council, to come a second time to the rescue of a corporation whose fitness as a licensing authority had been thus impugned upon the testimony of experts.

